# Pulsus Alternans in Critical Aortic Stenosis: When the Left Ventricle Is Failing

**DOI:** 10.1016/j.jaccas.2024.102572

**Published:** 2024-10-02

**Authors:** Baudouin Bourlond, David Meier, Etienne Pruvot, Pierre Monney, Georgios Tzimas

**Affiliations:** Department of Cardiology, Lausanne University Hospital and University of Lausanne, Lausanne, Switzerland

**Keywords:** pulsus alternans, severe aortic stenosis

## Abstract

A 58-year-old man presented with worsening dyspnea. Electrocardiogram showed variation in T-wave amplitude occurring every other beat. Transthoracic echocardiography revealed a severe aortic stenosis with beat-to-beat variation in stroke volume, suggestive of pulsus alternans. Recognition of pulsus alternans is important because it is considered a marker of poor prognosis.

## Graphical Abstract

**Figure:**
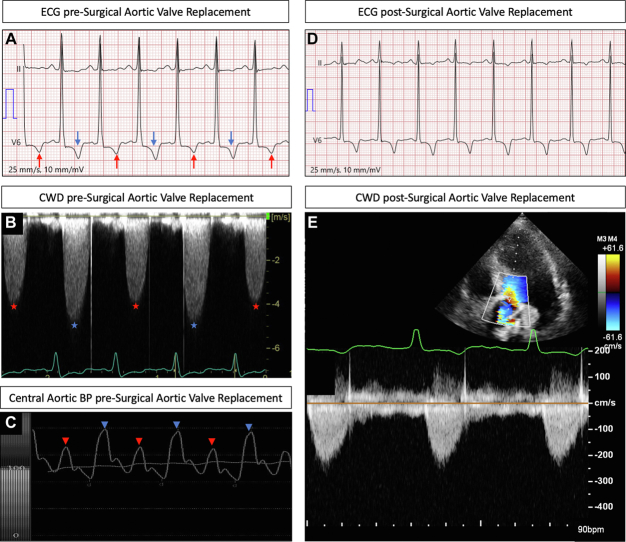

A 58-year-old patient, with no prior medical history, was admitted to our hospital because of progressively worsening dyspnea. Vital signs were as follows: blood pressure of 153/101 mm Hg, pulse of 104 beats/min; oxygen saturation of 92% (room air), and respiratory rate of 16 breaths/min. Physical examination revealed a high-pitched midsystolic aortic murmur with no sign of heart failure. Laboratory findings showed normal levels of inflammatory markers and hemoglobin. N-terminal pro–B-type natriuretic peptide was elevated (2,600 ng/L), and high-sensitivity T-troponin was slightly increased (45 ng/L). Electrocardiogram (ECG) and transthoracic echocardiogram (TTE) were performed (Figure 1). What is the most probable cause for the ECG and TTE findings?

1) Electrical alternans
2) Pulsus paradoxus
3) Pulsus alternans
4) T-wave alternans

**Figure 1:**
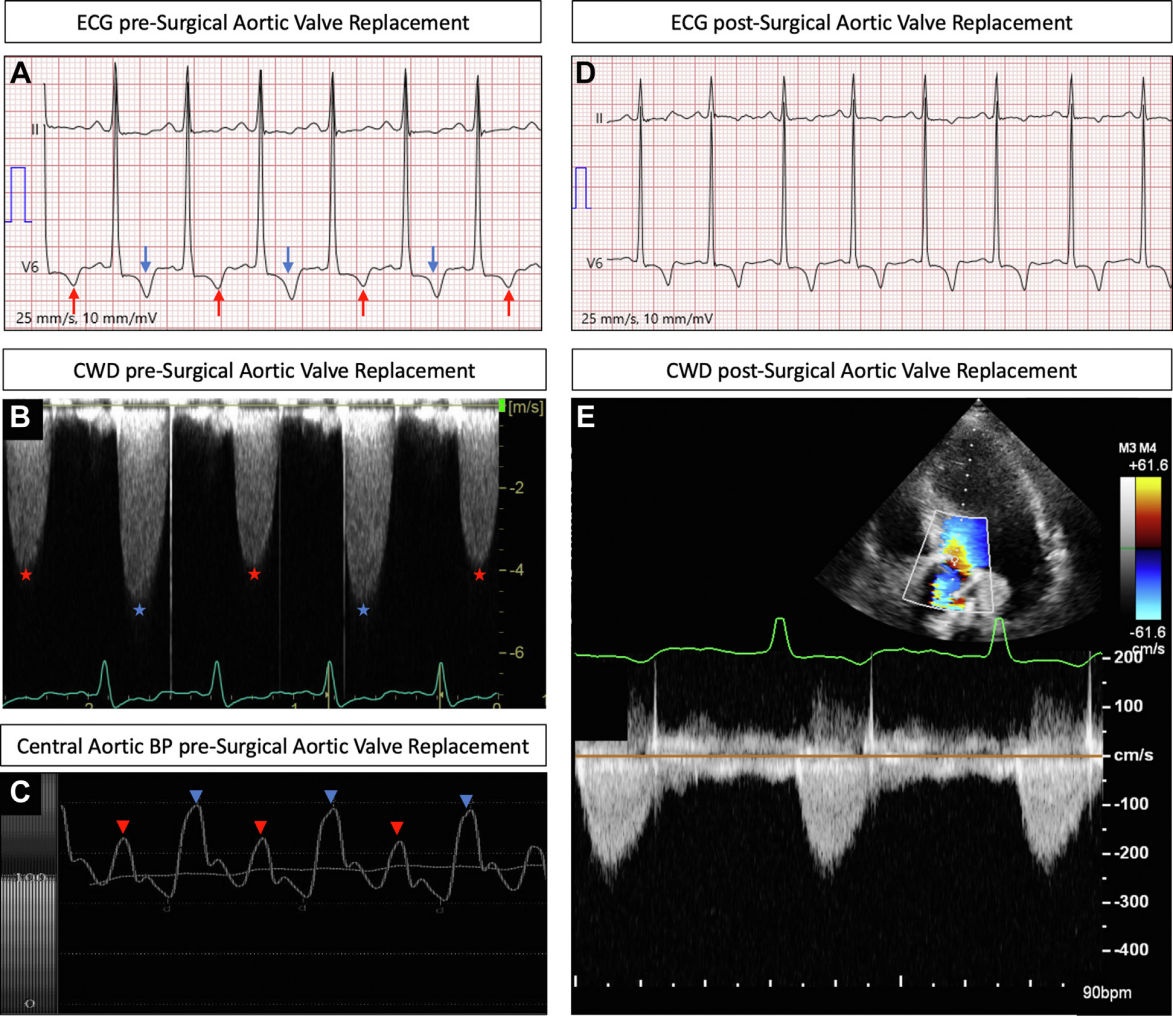
ECG, TTE, and Central BP Findings Pre-SAVR (A) ECG depicting variations in T-wave amplitude on each R-R interval (red and blue arrows). (B) Variation of peak transvalvular aortic velocity during each cardiac cycle observed on TTE (blue and red stars). (C) Central arterial BP variation measured from an intra-aortic catheter (red and blue triangles). (D) ECG showing no variation of T-wave amplitude. (E) TTE showing no variation on peak transvalvular aortic velocity post-SAVR. BP = blood pressure; CWD = continuous-wave Doppler; ECG = electrocardiogram; SAVR = surgical aortic valve replacement; TTE = transthoracic echocardiography.

## Discussion/Rationale

The ECG revealed a sinus rhythm with normal intervals and variation in T-wave amplitude occurring on a beat-to-beat basis (Figure 1A). TTE showed a bicuspid aortic valve with severe normal-flow high-gradient aortic stenosis (mean gradient: 60 mm Hg; aortic valve area: 0.8 cm^2^) and left ventricular global hypokinesia with reduced ejection fraction of 35%. Doppler interrogation revealed marked beat-to-beat variation of peak transvalvular aortic velocity (Figure 1B). Central arterial blood pressure showed alternating low- and high-amplitude waveforms suggestive of pulsus alternans (Figure 1C). Given the concomitant aortic root dilatation, the patient underwent a Bentall procedure with an INSPIRIS RESILIA 27 mm (Edwards Lifesciences). The postoperative period was uneventful, and the patient was discharged from the hospital 6 days after surgery. ECG and TTE (Supplemental Material) performed on the fifth day postsurgery showed no variation in T-wave amplitude and peak transvalvular aortic velocity (Figures 1D and 1E), respectively, and revealed an ejection fraction of 50%. The resolution of pulsus alternans after the aortic valve replacement supports the diagnosis of severe aortic stenosis with afterload mismatch as the cause of pulsus alternans.

Pulsus alternans is a rare hemodynamic condition characterized by beat-to-beat oscillations in cardiac muscle contraction at a steady heart rate and is accounted for by 2 main proposed mechanisms.^1^ The first hypothesis is based on the Frank-Starling relationship and posits that a weak beat resulting from left ventricular contractile failure and low stroke volume alternates with a strong beat resulting from an increased end-diastolic volume and myocardial stretch for the following cardiac cycle, which will transiently increase myocardial inotropy. The second hypothesis suggests a beat-to-beat alternation in myocardial contractility, attributed to abnormal calcium handling by cardiac myocytes.

Pulsus paradoxus is defined by an inspiratory (but not beat-to-beat) fall in systolic blood pressure >10 mm Hg, typically associated with cardiac tamponade or constrictive pericarditis. Electrical alternans is characterized by alternating QRS amplitudes observed in one or multiple ECG leads, typically associated with large pericardial effusion.^2^ T-wave alternans is an observed ECG finding of beat-to-beat alternation in T-waves morphology and or amplitude, commonly associated with a prolonged QT interval and an increased risk of malignant cardiac arrhythmias.^3^

This case illustrates that the hemodynamic impairment of pulsus alternans can also be visible on ECG and is not to be confused with the aforementioned differential diagnoses, which have different underlying pathophysiology and subsequent treatment.

## Funding Support and Author Disclosures

The authors have reported that they have no relationships relevant to the contents of this paper to disclose.

## Appendix

Video 1 Three-Chamber View on Transthoracic Echocardiography Showing a Severe Calcified Aortic Valve

Video 2 Three-Chamber View with Color Doppler on Transthoracic Echocardiography Showing Severe Eccentric Aliasing Through the Aortic Valve

Video 3 Three-Chamber View on Transthoracic Echocardiography Post Bentall Procedure Showing the Bioprosthetic Aortic Valve

Video 4 Three-Chamber View with Color Doppler Post-Bentall Procedure on Transthoracic Echocardiography Showing No Aliasing Through the Bioprosthetic Aortic Valve
