# Supplementary material for: Pulsus Alternans in Critical Aortic Stenosis: When the Left Ventricle Is Failing
Source: JACC Case Rep. 2024 Oct 2;29(19):102572. doi: 10.1016/j.jaccas.2024.102572 (PMC11522733; doi:10.1016/j.jaccas.2024.102572)

**Supplemental Appendix**

# Echocardiography findings:

Quantitative assessment of the mean gradient by transthoracic echocardiography (TTE) revealed a high and mean gradient at high beat pressure of 96 mmHg and 60 mmHg, respectively (**Supplemental Figure 2**, **Panel C & A**, respectively). The high and mean gradient at low beat pressure was 64 mmHg and 39 mmHg, respectively (**Supplemental Figure 1, Panel D & B**, respectively).

The pulsed wave Doppler in the LVOT showed a stroke volume and indexed stroke volume at high beat pressure of 83 ml and 37 ml/m2, respectively. The stroke volume and indexed stroke volume at low beat pressure was 58 ml and 26 ml/m2, respectively.

The VTI LVOT at high and low beat pressure was 15,2 cm and 10,7 cm, respectively.

The filling pressures were elevated, as estimated by echocardiography (E/A 0.6, E/e’ 14,1, TR velocity 3,1m/s, LA volume 45ml/m2).

## Supplemental Figure 1:

Volume rendering image (A) and maximum intensity projection image (B) showing the extent of the aortic valve calcification


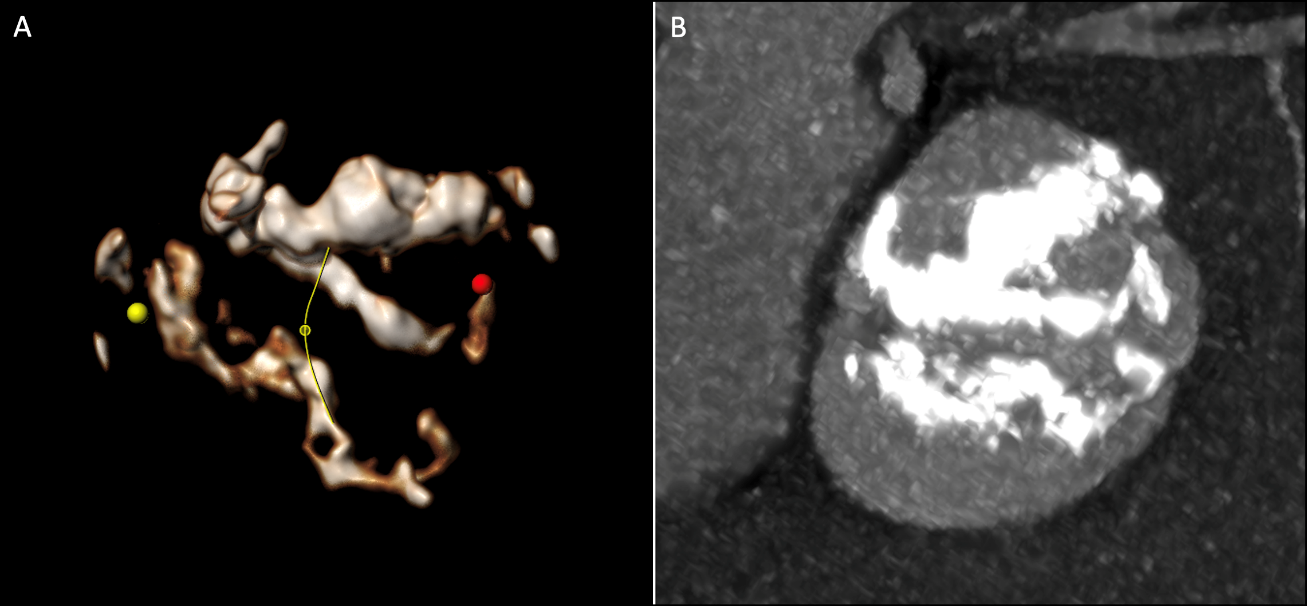


## Supplemental Figure 2:

TTE revealed a high and mean gradient at high beat pressure of 96 mmHg and 60 mmHg, respectively (Panel C & A). The high and mean gradient at low beat pressure was 64 mmHg and 39 mmHg, respectively (Panel D & B).


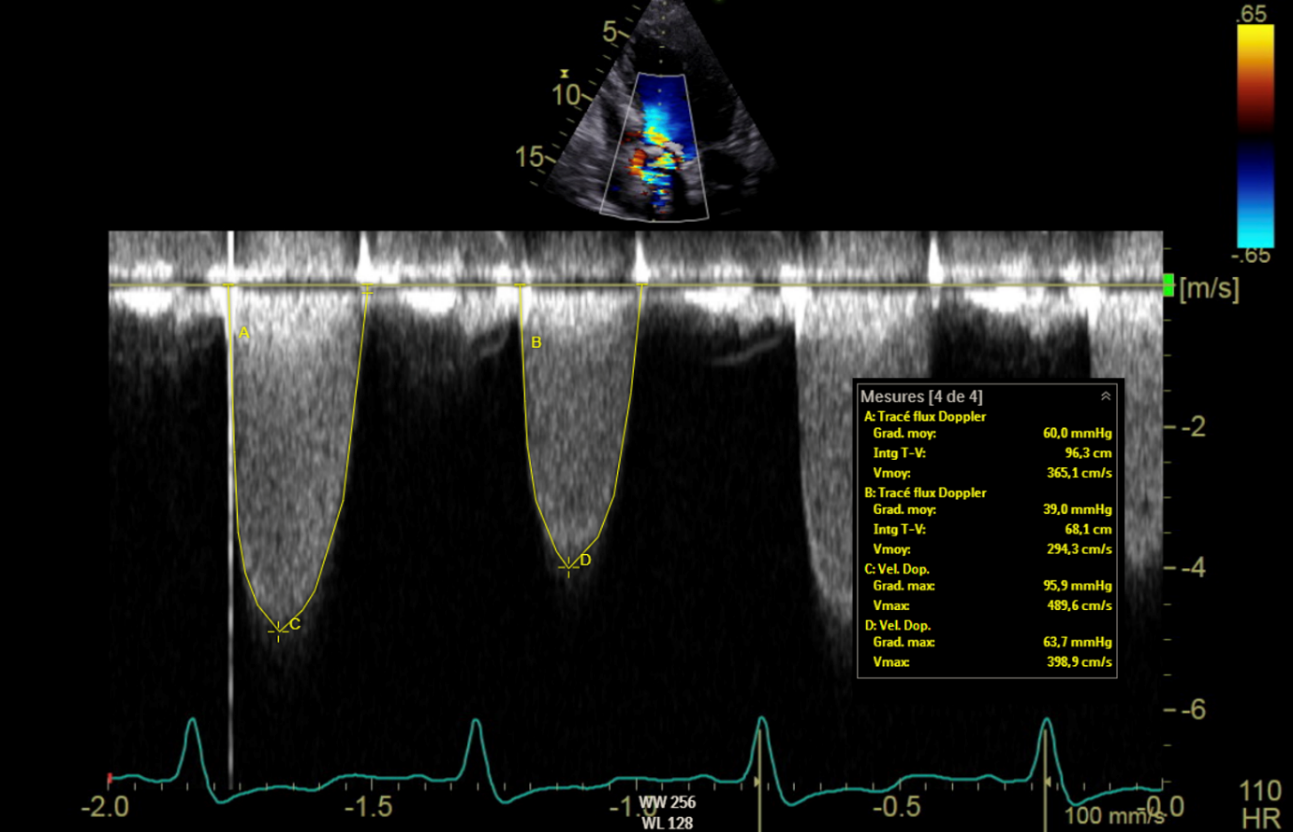

Supplement: Supplemental Material [file mmc5.docx]
